# Spatial expression pattern of serine proteases in the blood fluke *Schistosoma mansoni* determined by fluorescence RNA in situ hybridization

**DOI:** 10.1186/s13071-021-04773-8

**Published:** 2021-05-22

**Authors:** Lenka Ulrychová, Pavel Ostašov, Marta Chanová, Michael Mareš, Martin Horn, Jan Dvořák

**Affiliations:** 1 Institute of Organic Chemistry and Biochemistry, The Czech Academy of Sciences, Flemingovo n. 2, 16610 Prague, Czech Republic; 2 Department of Parasitology, Faculty of Science, Charles University, Viničná 7, 12844 Prague 2, Czech Republic; 3 Biomedical Center, Faculty of Medicine in Pilsen, Charles University, Alej Svobody 1655/76, 32300 Pilsen, Czech Republic; 4 Institute of Immunology and Microbiology, First Faculty of Medicine, Charles University and General University Hospital in Prague, Studničkova 2028/7, 12800 Prague, Czech Republic; 5 Department of Zoology and Fisheries, Centre of Infectious Animal Diseases, Faculty of Agrobiology, Food and Natural Resources, Czech University of Life Sciences in Prague, Kamýcká 129, 16500 Prague 6, Czech Republic

**Keywords:** Platyhelminthes, Blood fluke, *Schistosoma mansoni*, mRNA detection, Transcript, Fluorescence RNA in situ hybridization, Serine proteases

## Abstract

**Background:**

The blood flukes of genus *Schistosoma* are the causative agent of schistosomiasis, a parasitic disease that infects more than 200 million people worldwide. Proteases of schistosomes are involved in critical steps of host–parasite interactions and are promising therapeutic targets. We recently identified and characterized a group of S1 family *Schistosoma mansoni* serine proteases, including SmSP1 to SmSP5. Expression levels of some SmSPs in *S. mansoni* are low, and by standard genome sequencing technologies they are marginally detectable at the method threshold levels. Here, we report their spatial gene expression patterns in adult *S. mansoni* by the high-sensitivity localization assay.

**Methodology:**

Highly sensitive fluorescence in situ RNA hybridization (FISH) was modified and used for the localization of mRNAs encoding individual SmSP proteases (including low-expressed SmSPs) in tissues of adult worms. High sensitivity was obtained due to specifically prepared tissue and probes in combination with the employment of a signal amplification approach. The assay method was validated by detecting the expression patterns of a set of relevant reference genes including SmCB1, SmPOP, SmTSP-2, and Sm29 with localization formerly determined by other techniques.

**Results:**

FISH analysis revealed interesting expression patterns of SmSPs distributed in multiple tissues of *S. mansoni* adults. The expression patterns of individual SmSPs were distinct but in part overlapping and were consistent with existing transcriptome sequencing data. The exception were genes with significantly low expression, which were also localized in tissues where they had not previously been detected by RNA sequencing methods. In general, SmSPs were found in various tissues including reproductive organs, parenchymal cells, esophagus, and the tegumental surface.

**Conclusions:**

The FISH-based assay provided spatial information about the expression of five SmSPs in adult *S. mansoni* females and males. This highly sensitive method allowed visualization of low-abundantly expressed genes that are below the detection limits of standard in situ hybridization or by RNA sequencing. Thus, this technical approach turned out to be suitable for sensitive localization studies and may also be applicable for other trematodes. The results suggest that SmSPs may play roles in diverse processes of the parasite. Certain SmSPs expressed at the surface may be involved in host–parasite interactions.

**Graphic abstract:**

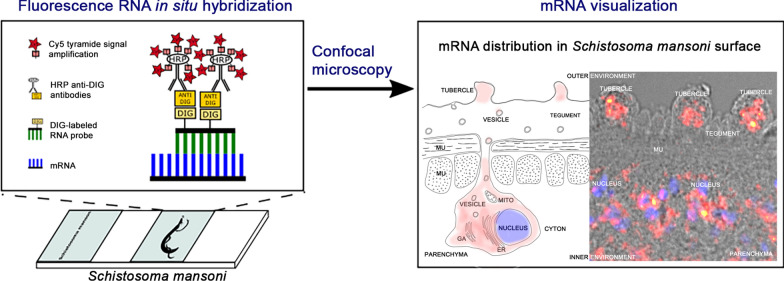

**Supplementary Information:**

The online version contains supplementary material available at 10.1186/s13071-021-04773-8.

## Background

Human schistosomiasis is a chronic infectious disease affecting more than 200 million people in 76 countries, mostly in tropical and subtropical areas, caused by flukes of the genus *Schistosoma* [1]. Current treatments rely on one drug, praziquantel, and no effective vaccine has yet been developed [2]. Several schistosome species infect humans, with three of the most abundant species being *Schistosoma haematobium*, which causes urinary schistosomiasis, and *Schistosoma japonicum* and *Schistosoma mansoni*, causing intestinal schistosomiasis [3]. Humans are infected by schistosome larvae called cercariae, which are released into freshwater by intermediate snail hosts. Cercariae penetrate human skin and subsequently develop into adult male or female worms in the host vascular system, where they produce hundreds of eggs per day [4]. Morbidity arises from immunopathological reactions to and entrapment of schistosome eggs in various tissues [5].

The genome of *S. mansoni* was sequenced, and numerous genes were identified or predicted [6]. In the post-genomic era, however, it is vital to elucidate functions of these genes. Quantification and spatial distribution of RNA transcripts in individual cells of the whole multicellular organism helps to narrow potential functions of individual genes [7–9]. RNA in situ hybridization represents a powerful tool to investigate the spatial distribution of gene transcripts [7, 10]. Hybridization methods are based on the binding of an individual RNA target with specifically designed complementary RNA probes labeled with a reporter molecule, such as digoxigenin (DIG). Such probes can then be detected by specific antibodies recognizing this molecule. These antibodies are conjugated with enzymes allowing visualization of the probe via fluorescent or colored staining. However, detection and visualization of low-abundantly expressed transcripts is challenging in multicellular organisms such as schistosomes with diverse cellular and tissue organization.

Blood flukes of the genus *Schistosoma* are unique among the trematodes because they have evolved separate sexes [11]. They are acoelomates, animals without a body cavity except for the gut and reproductive organs (testes, vitellaria, ovary, and oviduct). The interior body is filled with mesodermal tissue called parenchyma, which is composed of diverse cell subtypes such as stem cells [12], cells derived for example from neuro-excretory systems [13, 14], tegumental cell bodies [14, 15], and other yet undefined cell types with unknown functions. The entire surface of adult schistosomes is covered by the syncytial layer called the tegument [16].

Serine proteases (SPs) are key virulence factors for many parasitic helminths; they are critical for parasite invasion, migration, nutrition, and reproduction, and they facilitate adaption to and evasion from the host's physiological and immune responses [17–19]. Recently, we found that the S1 family of serine proteases of *S. mansoni* (SmSPs) significantly contributed to proteolytic activities detected in excretory/secretory (E/S) products of blood-dwelling developmental stages [20]. We uncovered a repertoire of SmSPs (designated SmSP1 to SmSP5) by performing a series of genomic, transcriptomic, proteolytic, and phylogenetic analyses [21], and described the major SmSP, SmSP2, at the protein level [22].

In this work, we used the fluorescence in situ hybridization (FISH) technique based on a detection of DIG-labeled RNA probes by anti-DIG antibody conjugated with horseradish peroxidase (HRP) [23] to investigate RNA distribution in adult male and female *S. mansoni*. The technique was validated by detecting a set of target RNA transcripts with known localization, the digestive protease cathepsin B1 of *S. mansoni* (SmCB1) [24–27], the surface-associated prolyl oligopeptidase (SmPOP) [28], the tegumental tetraspanin SmTsp-2 [29–31], and Sm29, a membrane-bound glycoprotein found at the *S. mansoni* tegument [31–34]. A probe targeting a bacterial neomycin gene (*neo*), which is absent in the *S. mansoni* genome, was used as a negative control. The validated FISH technique was then used to determine the tissue distribution of transcripts of individual SmSP genes (SmSP1 to SmSP5) [18, 21]. The analysis revealed complex expression patterns of all SmSPs generally consistent with existing transcriptome sequencing data [35, 36]. Moreover, the method also enabled us to localize the genes with significantly low expression in tissues where they had not previously been detected by RNA sequencing (RNAseq) methods.

## Methods

### Schistosome material

*S. mansoni* (a Puerto Rican LSHTM strain) was maintained in the laboratory by cycling between the intermediate snail hosts, *Biomphalaria glabrata*, and outbred ICR (CD-1) mice as definitive hosts. Infective larvae (cercariae) were shed by light stimulation from infected snails placed in bottled drinking water. Adult female mice were infected by immersing their feet and tail into 50 mL of water containing approximately 300 cercariae for 45 min. Six weeks post-infection, mice were over-anesthetized by an intraperitoneal injection of ketamine (Narkamon 5%—1.2 mL/kg body weight) and xylazine (Rometar 2%—0.6 mL/kg body weight), and the worms were recovered from the hepatic portal system by transcardial perfusion with RPMI 1640 medium (Sigma-Aldrich) as described previously [20, 22, 37].

### Isolation of mRNA and cDNA synthesis

Collected adult worms were washed three times with 50 mL phosphate-buffered saline (PBS) and re-suspended in 500 µL of the Trizol reagent (Thermo Fisher), and RNA was isolated as described previously [38]. Single-stranded cDNA was synthesized from total RNA by SuperScript III reverse transcriptase (Thermo Fisher) and an oligo(dT)_23_ primer according to the manufacturer’s protocol. The final cDNA product was purified using the QIAquick PCR purification kit (Qiagen) and stored at −20 °C.

### Probe production

Probes (600–1500 nucleotides) were designed to hybridize with the catalytic domain sequences of selected target gene transcripts. DNA templates for probe synthesis were amplified by polymerase chain reaction (PCR) from *S. mansoni* cDNA using gene-specific primers (Additional file 1: Table S1). The PCR products were cloned into the pGEM-T Easy vector (Promega), and the cloned sequences were verified by DNA sequencing. Constructs were linearized by restriction enzymes (NEB) selected based on insert orientation within the construct. Linearized plasmid DNA (1 µg) was used as a template to generate digoxigenin (DIG)-labeled probes by in vitro transcription using the DIG RNA labeling kit (SP6/T7) (Roche). The probes were transcribe using either SP6 or T7 RNA polymerases (final concentration 1 U per 1 µL) at 37 °C for 2 h according to the manufacturer’s protocol. DNA templates were removed by incubation with DNase I (final concentration 1 U per 1 µL) at 37 °C for 15 min. The reactions were terminated by the addition of EDTA (final concentration 0.02 M), and the probes were stored at −20 °C prior to use. DIG-labeled “antisense” Neo RNA, which is a component of the SP6/T7 DNA labeling kit (Roche), was used as a control probe. The DNA construct containing the Sm29 sequence was kindly provided by Christoph G. Grevelding (Justus Liebig University, Giessen, Germany). All hybridization probes were verified by the sequencing. The probe specificity was verified by BlastN analysis on the National Center for Biotechnology Information (NCBI) database. Sequences shared 100% identity with the studied genes and showed no significant similarities to other genes in the organism [35], including those used in our study.

### Tissue preparation for fluorescence in situ hybridization

Following perfusion, *S. mansoni* couples were separated on ice by gentle prodding with a brush, washed three times in 50 mL of PBS, and fixed with boiling 4% formaldehyde solution (Sigma-Aldrich). Worms were left to cool to 25 °C, incubated at 25 °C for 90 min, and dehydrated by incubation in a series of increasing ethanol concentrations (25%, 50%, 70%, 90%, 96%, 100% v/v at 25 °C for 5 min each). Incubation with 100% ethanol was performed twice for 5 min. Subsequently, males were incubated in methyl benzoate (Sigma-Aldrich) at 25 °C for 45 min, females for 20 min. Worms were washed twice at 25 °C for 5 min each in benzene (Sigma-Aldrich), which was then exchanged three times with 60 °C hot paraffin (Paraplast X-TRA, Leica). All incubation and washing steps were performed in approximately 50 mL of appropriate solution. After the last wash, worms were incubated in 20 mL paraffin (Leica) at 60 °C for 2 h and then embedded in paraffin blocks. Sections (6 µm) were prepared on a microtome (Shandon Finesse^®^ ME+) and applied to X-TRA adhesive glass slides (Leica).

### Pre-hybridization tissue treatment

Slides with fixed tissue sections (6 µm) were de-paraffinized by two 5-min washes in xylene followed by two 5-min washes in 100% ethanol. Sections were rehydrated by incubation in a series of decreasing ethanol concentrations (100%, 96%, 90%, 70%, 50%, 25% v/v) at 25 °C for 5 min each, followed by a wash in diethyl-pyrocarbonate (DEPC, Sigma-Aldrich)-treated water for 5 min. To minimize background quench by endogenous peroxidases and improve cell permeability, slides were incubated in 0.2 N HCl at 25 °C for 20 min, followed by incubation in 0.01 M sodium citrate, pH 6.0, in a boiling water bath for 15 min. Slides were then cooled to 25 °C for 30 min, incubated in 0.2% glycine for 5 min, followed by incubation in ice-cold 20% acetic acid for 15 s and PBS at 25 °C for 5 min. Finally, the slides were incubated in 20% glycerol at 25 °C for 15 min and briefly rinsed with 2× saline sodium citrate buffer (SSC, Sigma-Aldrich). All incubation and washing steps were performed in 150 mL of appropriate solution.

### RNA in situ hybridization

To denature secondary RNA structures within the tissues, sections were preheated to 70 °C for 10 min prior to hybridization and briefly cooled on an ice-cold metal plate. One hundred microlitres of hybridization mixture A [5× SSC, 1x PBS, 0.1% torula yeast RNA (Sigma-Aldrich)] containing RNA probe (0.5–5 ng/mL) was heated to 70 °C to denature secondary RNA structures. The mixture was then briefly cooled on ice, immediately mixed with 160 µL of hybridization solution B [50% formamide, 10% dextran sulfate molecular weight 4000 (Sigma-Aldrich) and 1% Tween 20], applied to sections, and covered with a coverslip. All samples were then hybridized in a moisture chamber at 42 °C for 16 h.

### Post-hybridization treatment

After hybridization, the slides were washed in 2x SSC with 0.1% Tween 20 at 42 °C for 15 min, followed by washes in 1x SSC, 0.5x SSC, and 0.1x SSC, each at 25 °C for 15 min. The slides were then washed twice for 5 min with MAB buffer (0.1 M maleic acid, pH 7.5, 0.15 M NaCl) at 25 °C and incubated in 4% blocking solution [4% heat-inactivated horse serum (Sigma-Aldrich) in MAB buffer] for 30 min. The DIG-labeled probes hybridized with tissue RNA were labeled by incubation with anti-digoxigenin antibody conjugated with horseradish peroxidase (anti-DIG-HRP antibody, Perkin Elmer) diluted 1:500 in 2% blocking solution (2% heat-inactivated horse serum in MAB buffer) at 37 °C for 2 h. Excess antibody was then removed by three washes in MAB at 25 °C for 10 min. All incubation and washing steps were performed in 150 mL of appropriate solution. Hybridized probes labeled with DIG-HRP antibodies were visualized by the Tyramide Signal Amplification (TSA) system with the Cyanine Plus 5 Tyramide Reagent fluorescence system (Perkin Elmer) according to the manufacturer’s protocol. Briefly, the slides were washed three times with 150 mL TNT buffer (0.1 M Tris–HCl, pH 7.5, 0.15 M NaCl, 0.05% Tween 20) for 5 min each, and incubated in a moisture chamber with Cyanine Plus 5 dye diluted 1:50 in 500 µL of 1× Plus Amplification Diluent for 10 min. After three washes with 150 mL TNT buffer for 5 min, sections were rinsed in DEPC-water and mounted in ProLong Diamond Antifade reagent containing DAPI (Thermo Fischer).

### Microscope observation

Fluorescent signals were detected using an Olympus IX83 fluorescence microscope (Olympus) equipped with a pco.edge 5.5 camera. To define the background signal threshold, samples underwent FISH procedure without probe, or hybridization with the control probe carrying the sequence of a bacterial neomycin-resistance gene (*neo*) which is not present in the schistosome genome. Microscopy images were processed using Fiji software [39]. Images were collected from three parts of adult worms: the anterior head region (females: from ventral sucker to the anterior margin of ovaries; males: extending from the anterior extremity of the schistosome to the ventral sucker), the middle region (females: containing mature and immature oocytes and oviduct; males: containing testis), and the posterior hind region (females: containing vitellaria and gut; males: the body part located posterior to the margin of testes and focusing on the parenchyma, tegument, and gut). This division is very coarse and used for simplification; some organs may occur in several regions. Moreover, obtaining a representative image of the anterior region of the female (head with esophagus) is generally problematic because fixation always causes some degree of contraction and distortion, and sectioning is difficult. To verify reproducibility, all experiments including probe production, tissue preparation, and FISH procedure were carried out in at least two independent experiments.

## Results

### Validation of fluorescence RNA in situ hybridization (FISH) for the tissues of *S. mansoni* adults

We used FISH based on digoxigenin (DIG)-labeled RNA probes [23] to localize individual RNA molecules in *S. mansoni* adult males and females. Initially, the FISH assay was validated by localization of a set of relevant control genes with a known localization [determined using other localization techniques, e.g. immunolocalization or whole-mount in situ hybridization (WISH)]. The genes used for validation were digestive protease SmCB1 [40], surface-localized protease SmPOP [28], and tegumental proteins SmTsp-2 [41] and Sm29 [33].

**Fig. 1:**
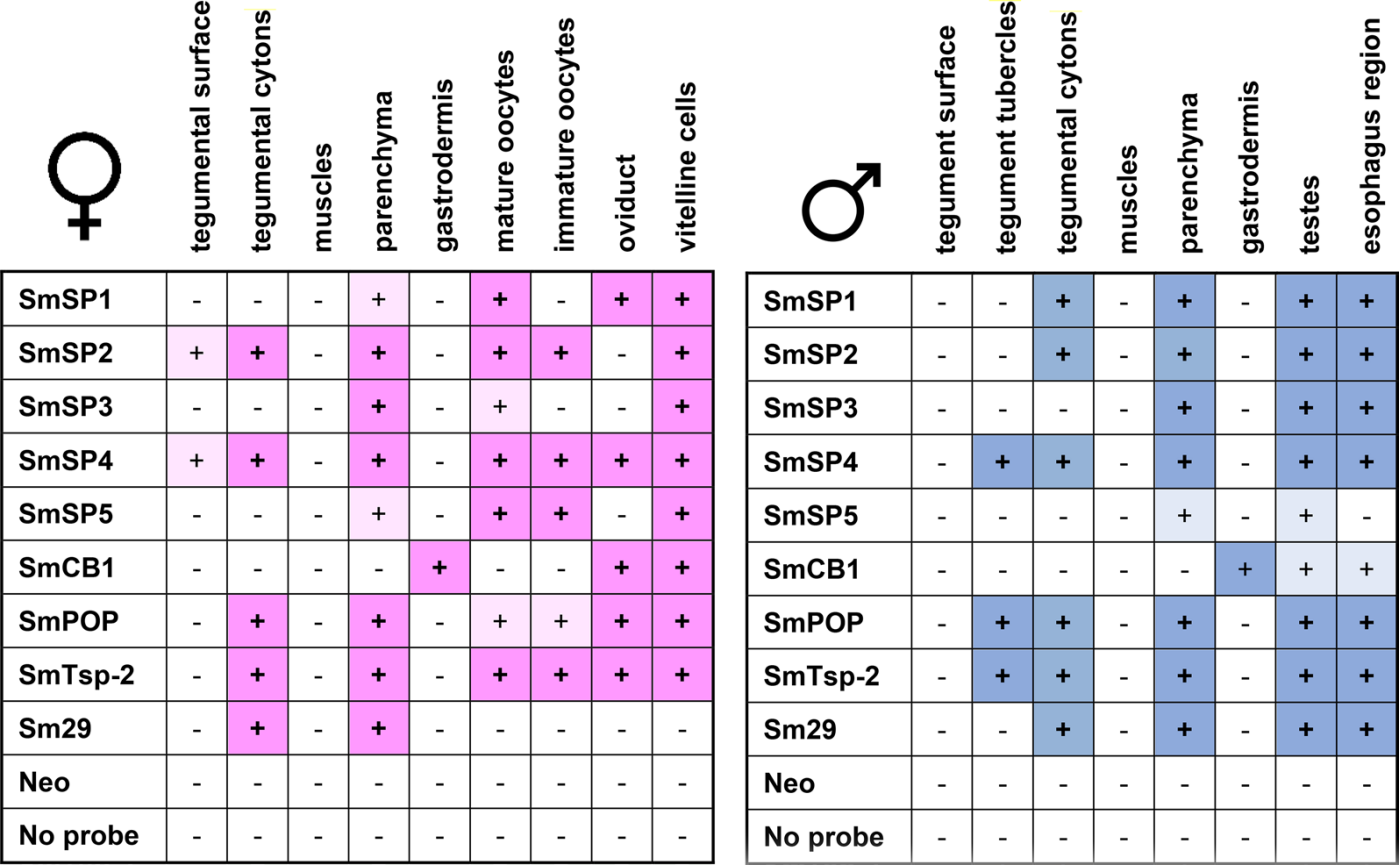
An overview of localization of gene sense transcripts in *S. mansoni* adult male and female tissues using FISH. Transcripts whose localizations were observed in the given tissue are highlighted in pink (females) or blue (males). Light shades represent a faint positive signal for a given transcript. Localization in the esophagus region was possible only in the sections of adult males; obtaining a representative section of the smaller females’ head region (containing esophagus) is generally problematic

The localization results obtained for individual target RNAs are summarized in Fig. 1. Generally, transcripts of the genes were localized by FISH in the same tissues of adult males or females as previously described. Due to the high sensitivity of the technique, additional new localizations of some transcripts were found. The transcript profiles for SmCB1, SmPOP, SmTsp-2, and Sm29 also corresponded to previous RNAseq data obtained for *S. mansoni* adults and their gonads (http://schisto.xyz/; [35, 36]).

**Fig. 2:**
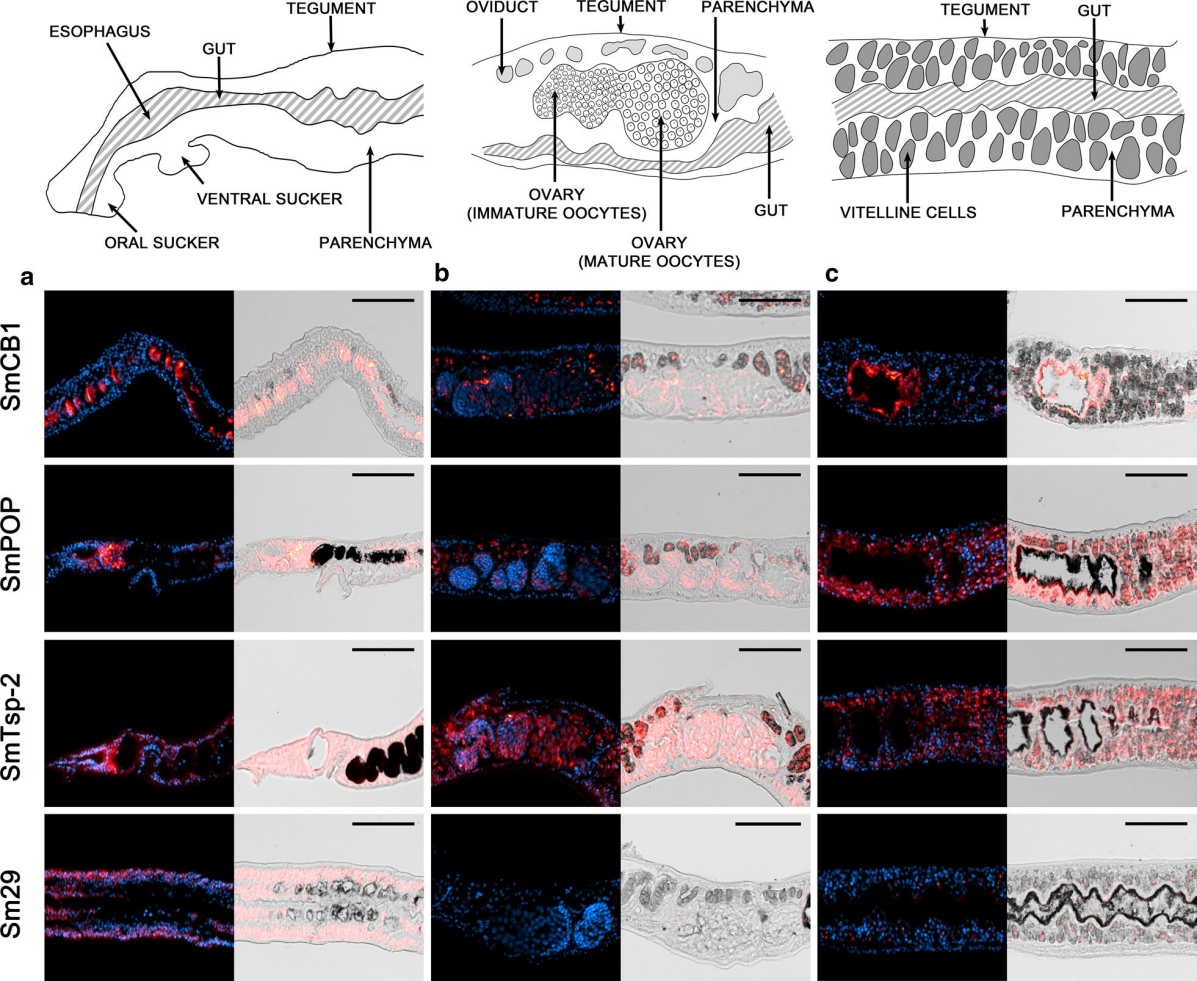
Localization of mRNAs encoding SmCB1, SmPOP, SmTsp-2, and Sm29 in adult *S. mansoni* females using FISH. Semi-thin (6 μm) sections of adult *S. mansoni* female worms were probed with DIG-labelled RNA probes designed to detect SmCB1, SmPOP, SmTsp-2, or Sm29 mRNAs. The probes hybridized with transcripts were visualized by tyramide amplification assay (red). Adult females were monitored in three parts: **a** an anterior part, **b** oviduct, mature and immature oocytes, and **c** vitellaria and gut. DAPI was used to label nuclear DNA (blue). The left columns show merged fluorescent channels; in the right columns, the fluorescent red signal is merged with differential interference contrast. **a** A strong signal of SmPOP, SmTsp-2; and Sm29 was detected in parenchymal cells of the anterior part of the female. **b** A strong fluorescent signal of SmTsp-2 and faint scattered SmPOP signals were observed in mature and immature oocytes; the oviduct is rich in SmCB1, SmPOP, and SmTsp-2 transcripts. **c** SmPOP and SmTsp-2 mRNAs are strongly expressed in vitellaria, while mRNAs of SmCB1 and Sm29 are not shown. **a**–**c** Transcripts of SmCB1 are present in the gut throughout the whole worm. The scale bars represent 100 µm

**Fig. 3:**
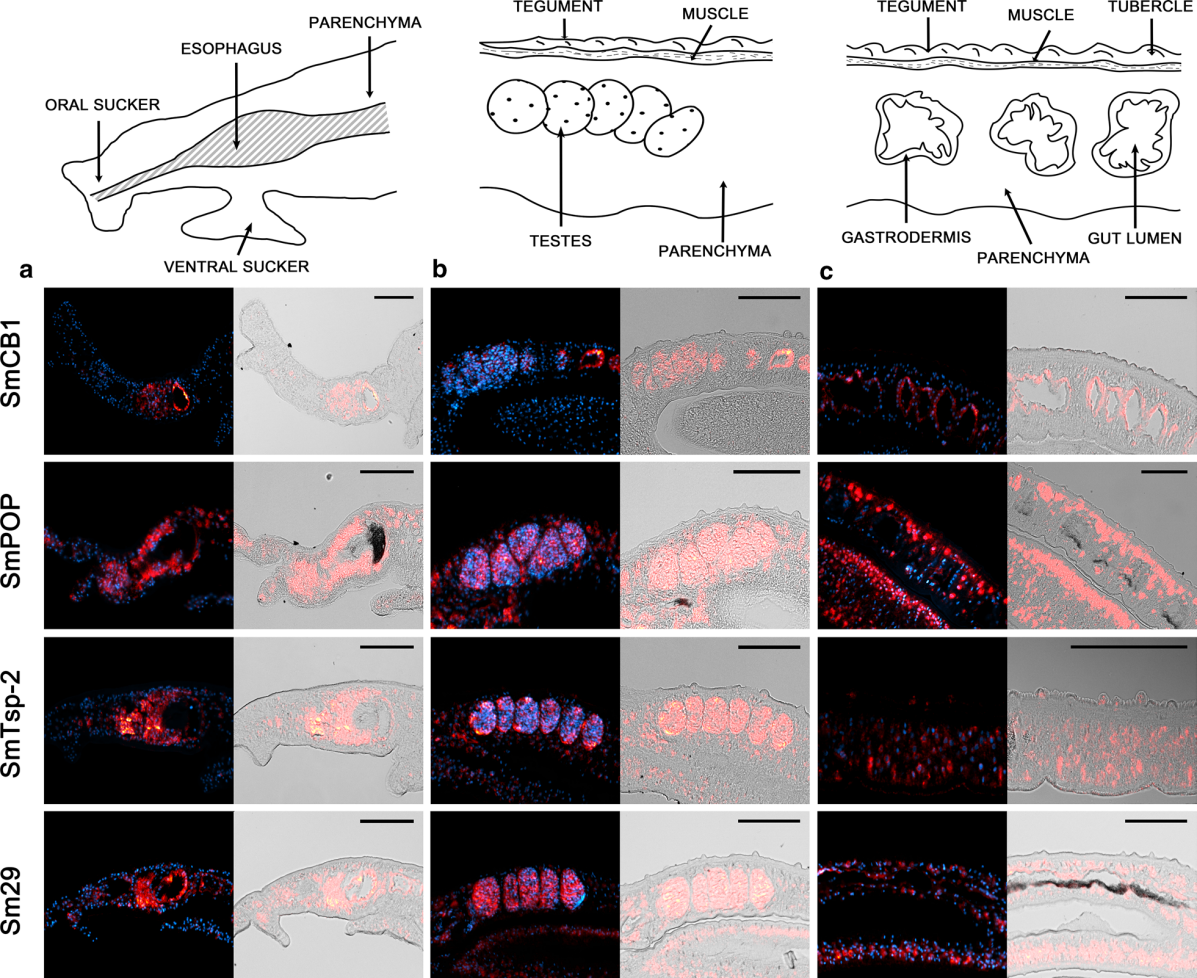
Localization of mRNAs encoding SmCB1, SmPOP, SmTsp-2, and Sm29 in adult *S. mansoni* males using FISH. Semi-thin (6 μm) sections of adult *S. mansoni* male worms were probed with DIG-labelled RNA probes designed to detect SmCB1, SmPOP, SmTsp-2, or Sm29 mRNAs. The probes hybridized with transcripts were visualized by tyramide amplification assay (red). Adult males were monitored in three parts: **a** a head part, **b** testes, and **c** a posterior part with the focus on parenchyma, tegument and gut. DAPI was used to label nuclear DNA (blue). The left columns show merged fluorescent channels; in the right columns, the fluorescent red signal is merged with differential interference contrast. Transcripts of SmPOP, SmTsp-2, and Sm29 were detected in and around the esophagus, in the testes, and in parenchymal cell subtypes. SmPOP and SmTsp-2 were also detected in tegumental cells and in tegumental tubercles. SmCB1 is expressed strongly in the gut, weakly in parenchyma around the esophagus and testes. The scale bars represent 100 µm

Digestive SmCB1 was previously detected in the gastrodermis [40], and its activity was also demonstrated by fluorescence histochemistry in the vitellaria [42]. We confirmed localization of the transcripts coding SmCB1 in the gastrodermis of females and males and in the vitellaria (Figs. 2 and 3). Additionally, in females, a significant signal was present in the oviduct and a faint signal was detected in the parenchyma surrounding mature oocytes (Fig. 2). In males, faint fluorescence was present in testicular cells and in the region of the esophagus (Fig. 3).

SmPOP is a protease that was immunolocalized to the tegument (including tubercles) and the parenchyma of adult parasites [28]. In males, transcripts of SmPOP were detected in the tegumental cytons and, surprisingly, in tegumental tubercles—morphological structures above the muscle layer (Additional file 2: Figure S1a)—which are not expected to contain RNA molecules and where RNA translation should therefore not occur. Transcripts of SmPOP were also detected in parenchyma, around the esophagus, and in the testes (Fig. 3). In females, SmPOP was distributed in the parenchyma throughout the whole body, a strong signal was detected in the oviduct and the vitellaria, and a faint signal was also detected in oocytes (Fig. 2).

SmTsp-2 and Sm29 are known as tegumental markers [29, 30, 33, 41, 43, 44]. Transcripts of SmTsp-2 were detected in the tegument of both males and females and in the parenchyma (Figs. 2 and 3). In females, transcripts were also detected in the ovary, vitellaria, and oviduct, and in males, around the esophagus and in the testes. Surprisingly, but in accordance with previous findings [14], SmTsp-2 transcripts were also localized in the tegumental tubercles (Additional file 2: Figure S1).

Contrary to SmTsp-2, transcripts of Sm29 had a distinct expression pattern. In females (Fig. 2), strong fluorescence was found only in the parenchyma and tegumental cytons in the anterior regions. In males, Sm29 mRNA was found in the testes, some parenchymal cell subtypes, in the esophageal area, and in the tegumental cytons but not in the tegumental surface layer and tubercles (Fig. 3, Additional file 2: Figure S1a). No signal was detected in the ovary, oviduct, or gut of either gender (Figs. 2 and 3).

Also, sense probes hybridizing to antisense gene transcripts that do not encode proteins were designed for genes of all transcripts studied (Additional file 3: Figures S2 and S3) and used in the FISH procedure. Only antisense transcripts of SmPOP and SmCB1 were found solely in the female oviduct. No antisense transcripts of these genes were detected in the tissues of the males.

Non-specific bindings and backgrounds were analyzed using two different approaches: (i) hybridization with a probe targeting a sequence-coding bacterial gene (*neo*) which is not naturally present in genomes of schistosomes, and (ii) hybridization procedure without any probe. No signals were detected after the hybridization procedure in either case (Additional file 3: Figure S4).

The validated and optimized FISH therefore represents a robust method for detection of RNA in sections of adult *S. mansoni*. This was documented by the precise localization of selected transcripts, and the result corresponded to previous transcript profiling as determined by other methods such as immunolocalization [28, 33, 40, 43] or differential RNAseq [35, 36].

### FISH revealed a diverse distribution of serine proteases SmSP1 to SmSP5 in adult *S. mansoni*

**Fig. 4:**
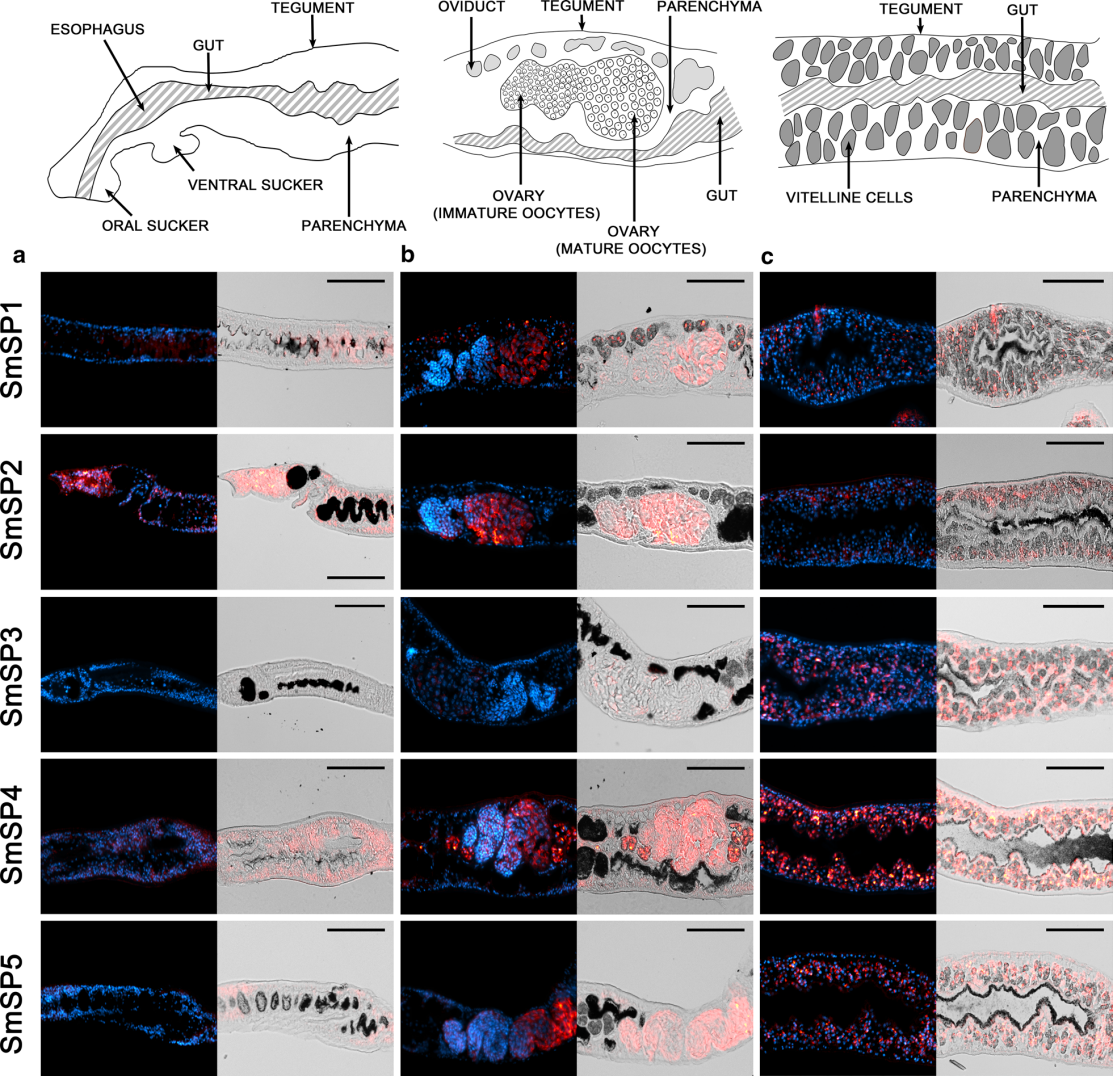
Localization of mRNA encoding SmSP1 to SmSP5 in adult *S. mansoni* females using FISH. Semi-thin (6 μm) sections of adult *S. mansoni* female worms were probed with DIG-labelled RNA probes designed to detect mRNAs of serine proteases SmSP1 to SmSP5. Probes hybridized with transcripts were visualized by tyramide amplification assay (red). Adult females were monitored in three parts: **a** an anterior part, **b** oviduct, mature and immature oocytes, and **c** vitellaria and gut. DAPI was used to label nuclear DNA (blue). The left columns show merged fluorescent channels; in the right columns, the fluorescent red signal is merged with differential interference contrast. **a** A strong signal of transcripts (red) belonging to SmSP2 and SmSP4 genes and less abundant signal of SmSP1 and SmSP5 was detected in parenchymal cells of the anterior part of the females. SmSP3 was not detected in this part. **b** Strong fluorescence of SmSP2, SmSP4, and SmSP5 was observed in mature and immature oocytes, while SmSP1 sense transcript is expressed exclusively in mature oocytes, and faint signal of SmSP3 was detected in ovary. SmSP1 and SmSP4 are also expressed in the oviduct. **c** Transcripts of all studied SmSPs were present in vitellaria but with different patterns of expression. A faint fluorescent signal of SmSP2 and SmSP4 transcripts is also detected in tegument. The scale bars represent 100 µm

**Fig. 5:**
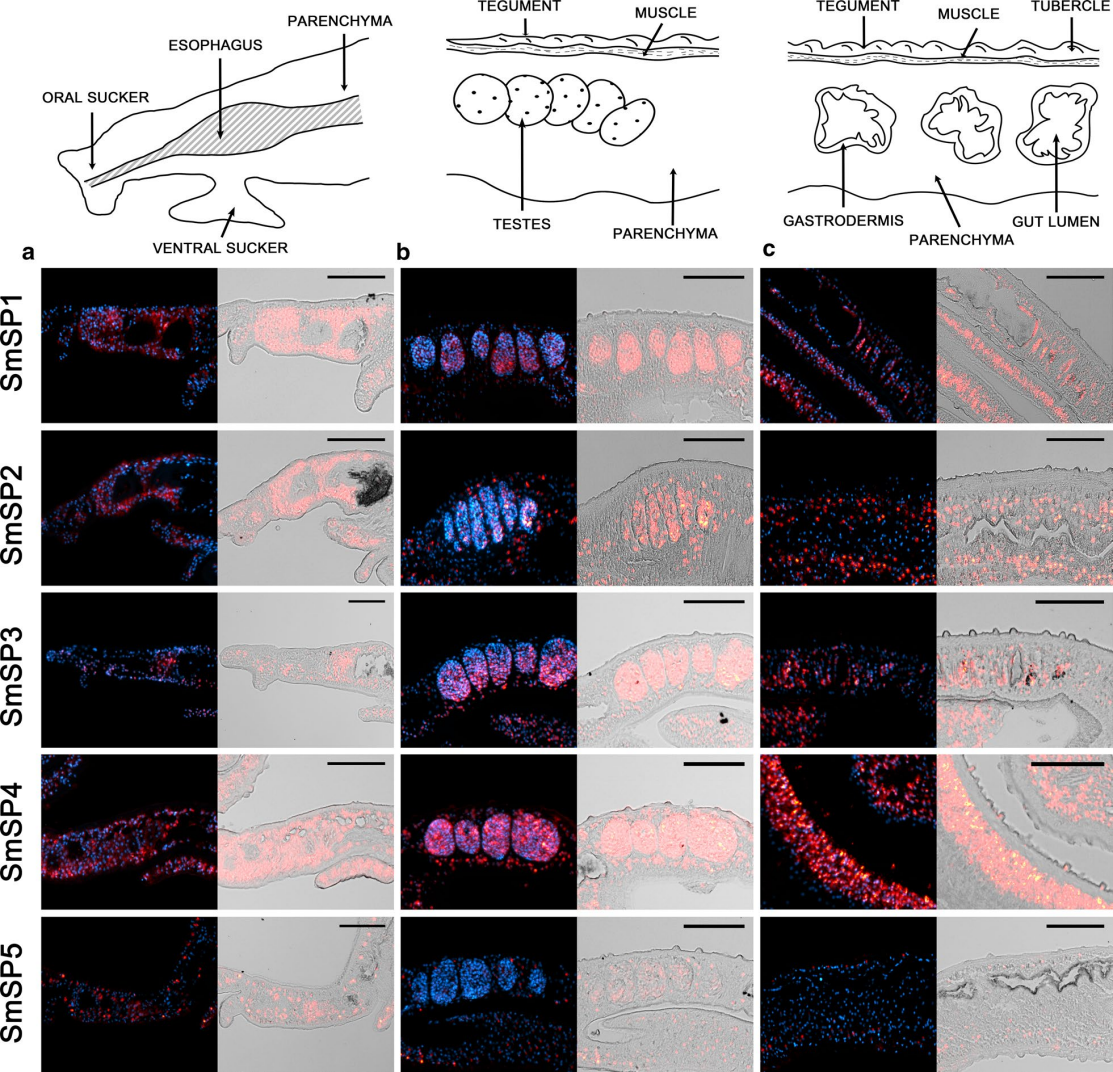
Semi-thin (6 μm) sections of adult *S. mansoni* male worms were probed with DIG-labelled RNA probes designed to detect mRNAs of serine proteases SmSP1 to SmSP5. Probes hybridized with transcripts were visualized by tyramide amplification assay (red). Adult males were monitored in three parts: **a** a head part, **b** testes, and **c** a posterior part with the focus on parenchyma, tegument, and gut. DAPI was used to label nuclear DNA (blue). The left columns show merged fluorescent channels; in the right columns, the fluorescent red signal is merged with differential interference contrast. **a** Except for SmSP5, transcripts of all SmSPs were identified in parenchyma around the esophagus. **b** Strong signal of SmSP1 to SmSP4 transcripts was detected in testes where only weak signal of SmSP5 mRNA was observed. **c** All SmSPs are present in parenchymal cell subtypes, however each with a different pattern. Compared to others, SmSP4 is abundant in parenchymal cells, in tegumental cytons, and also in tegumental tubercles. The scale bars represent 100 µm

The FISH technique was used to precisely localize transcripts encoding serine proteases SmSP1 to SmSP5 in adult *S. mansoni*. Results showing localization of SmSPs RNA are summarized in Fig. 1. Generally, transcripts of all SmSP1 to SmSP5 were detected in the parenchyma with different patterns. No expression of SmSPs was detected in the gastrodermis or the muscles. In other tissues, SmSPs showed diverse expression patterns that are described below (Figs. 4 and 5).

In females, SmSP1 transcripts were detected in the oviduct, vitellaria, and mature but not immature oocytes (Fig. 4). In males, SmSP1 genes were transcribed in the tegumental cytons, the area around the esophagus, and in the testes (Fig. 5). Low expression occurred in the parenchyma in both genders (Figs. 4 and 5).

SmSP2 transcripts were found in the esophagus and parenchyma throughout the bodies of both genders and also in the tegumental cytons, which agrees with previously described SmSP2 immunolocalization [22] (Figs. 4 and 5, Additional file 2: Figure S1a). In females, SmSP2 gene transcripts were also found in mature and immature oocytes, vitellaria, and in the tegumental surface above the muscle layer (Fig. 4); in males in testes (Fig. 5). No expression occurred in the tubercles or in the oviduct.

SmSP3 mRNA was found only in the parenchymal tissue surrounding the vitellaria in the posterior region of females, in the vitelline cells, and weakly in the mature oocytes (Fig. 4). Males expressed SmSP3 only in the esophagus region, parenchyma, and testes (Fig. 5). No fluorescence was detected in the immature oocytes, the oviduct, the tegumental surface, or the tegumental tubercles (Figs. 4 and 5).

SmSP4 was abundantly expressed in parenchymal cells and tegumental cytons in whole adult worms. SmSP4 transcripts were also detected in females in the tegumental surface, oviduct, mature and immature oocytes, and the vitelline cells (Fig. 4). Expression of SmSP4 in males occurred additionally in the testes and the tegumental tubercles (Fig. 5).

SmSP5 expression was revealed in mature and immature oocytes, and in the vitelline cells. Weak fluoresce was detected in the parenchyma, none in the oviduct (Fig. 4). In males, SmSP5 showed a different expression pattern compared to other SmSPs: a small number of parenchymal cells expressed SmSP5. In comparison with other SmSPs, only faint fluorescence was detected in the esophageal region and the testes, and no SmSP5 signal was detected in the tegument (Fig. 5).

As in the case of transcripts of proteins with known localizations (see above), sense probes for the detection of antisense transcripts of all SmSPs were designed and used to hybridize with the female and male dissected tissues. Only antisense transcripts of SmSP5 were found in the oviducts of females where no sense SmSP5 was expressed (Additional file 3: Figures S5 and S6).

FISH technology therefore allowed detection of the diverse localization of transcripts of all SmSP genes in adult schistosomes. The results showed their distinct distribution patterns within the tissues of males and females. A common feature for all SmSPs was the expression in parenchymal tissue; however, further expression patterns varied. None of these transcripts was detected in the gastrodermis or muscle layer.

## Discussion

Family S1 serine proteases (SPs) are crucial for successful parasitism by facilitating invasion, nutrient intake, and evasion of the host immune system, and modulation of the host physiology [17, 19]. However, information about SPs in *S. mansoni* (SmSPs) remains limited despite current discoveries [18, 22]. Our previous study demonstrated that SmSPs, designated SmSP1 to SmSP5, were differentially expressed among *S. mansoni* developmental stages [21]. Here, we used fluorescence in situ hybridization (FISH) to localize individual RNA molecules in *S. mansoni* adults in tissues including the esophagus, testis, ovary, vitellarium, and parenchymal and tegumental cells, including tubercles.

The FISH method was validated by detecting expression patterns for a set of transcripts of formerly described genes. We employed the previously established FISH protocol [45] by using (i) gentle tissue fixation and optimization of several pre- and post-hybridization steps with antigen retrieval, (ii) background minimization procedures, and (iii) amplification of the fluorescent signal by TSA [23]. The obtained results corresponded well with previous findings reported using other techniques: transcripts of digestive protease SmCB1 were localized in the gut, the expression of surface-localized SmPOP was detected in the parenchyma and tegument, and the mRNAs of tegumental SmTsp-2 and Sm29 were found in tegumental cytons or tegument [28, 33, 40, 41]. SmPOP and Sm29 transcripts were also localized in the vitellarium, which agrees with previous studies [28, 30, 33]. Lastly, the transcription profiles for SmCB1, SmPOP, SmTsp-2, and Sm29 were congruent with sequencing data obtained for *S. mansoni* [35, 36].

In addition to previous data, we discovered new localizations of some transcripts due to the high sensitivity of the FISH method. SmCB1 transcripts were detected in the vitellarium, whereas SmCB1 activity was previously detected by fluorescence histology only in oviducts and testes [42]. Localization of SmCB1 in vitellaria is in the line with expression of orthologous proteases from other parasitic flukes, *Fasciola gigantica* and *Eudiplozoon nipponicum*, in which cathepsin B expression was also detected in testes and vitelline cells [46, 47]. The function of these proteases in reproductive organs is as yet unknown; however, the authors hypothesized that cathepsin B may process eggshell or yolk protein vitellogenin precursors, as described in other organisms [48–50]. In addition to localization in the tegument, SmTsp-2 and Sm29 transcripts were found in parenchyma and testes, and SmTsp-2 also in the oviduct (Figs. 2 and 3). Tetraspanins stabilize extracellular vesicles [51], and these types of vesicles are known to be secreted as well by the specific types of cells into the lumen of the oviduct [52], which may explain the abundance of tetraspanin SmTsp-2 transcripts in such a highly dynamic organ structure.

Our previous research [21] and transcriptome sequencing data [9, 35, 36, 53] revealed that SmSP2 and SmSP4 are highly abundantly transcribed in adult schistosomes and, contrarily, expression levels of SmSP1, SmSP3, and SmSP5 are low. Highly sensitive FISH analysis employed in this paper revealed expression patterns of all SmSPs (including low-abundantly expressed ones) distributed in multiple tissues of adult schistosomes, except for more specific localization identified for SmSP3 in females and SmSP5 in males. All SmSPs were commonly detected only in parenchyma of both genders (Figs. 4 and 5), but individual SmSPs showed distinct expression patterns in different subtypes of parenchymal cells (Figs. 4 and 5), indicating their unique functional roles. Distribution of SmSP1 and SmSP2 transcripts in parenchymal cells and tegument agrees with protein immunolocalization data [22, 54]. Except for SmSP5, all studied genes localized within the anterior part of males in the head area containing the esophagus. Previously, this area was shown to be highly dynamic in gene transcription; more than 1000 genes were detected. Among them, SmSP2 was found to be upregulated twofold or more in this region in comparison with the whole worm body [55]. This agrees with our results, because strong signals of SmSP2 transcripts were detected in the parenchyma of the anterior body of both genders (Figs. 4 and 5). Detection of SmSP2 transcripts in the esophageal region also fits with our previous immunolocalization data [22].

Additional new localizations of several SPs were found in some reproductive organs of male or female worms. These organs are highly dynamic, comprising 7000 transcripts of genes upregulated twofold or more in reproductive organs compared to whole worm controls [36, 55]. Expression levels of particular genes are upregulated twofold or more after mating, and RNA storage is also frequently observed in gonadal cells [36, 56]. Additionally, mRNA stored in spermatozoa can be transported from males to females during sexual reproduction via sperm fluid into the oocyte [36, 57]. FISH revealed the presence of all SmSPs in gonads of both genders (Figs. 4 and 5). Contrarily, RNAseq showed (with exception of SmSP2) low or no SmSP transcripts in schistosome gonads [36]. Such divergence is probably based on distinct methodologies. RNAseq has a limited number of reads, reflecting a limited number of molecules that can be detected. Thus, low-abundant transcripts may be below the detection limit. On the other hand, FISH employed in this study has the capacity to detect every single RNA in the tissue due to amplification of the fluorescent signal by TSA (signal amplification up to 200fold), which has the ability to detect low-abundant transcripts which under normal circumstances would be part of the threshold. SmSPs may have a similar function reported for serine proteases in the gonads of several nematodes or flies: *Caenorhabditis elegans* or *Ascaris suum* employ serine proteases for spermatogenesis and sperm activation in the uterus [58, 59]; serine proteases of *Drosophila melanogaster* are thought to process peptides and activate enzymes inside the female reproductive ducts and mediate critical post-mating responses [60].

The entire surface of adult schistosomes is covered by the syncytial layer called the tegument [16]. In contrast to females*,* males have additional tegumental structures called tubercles [61]. The nucleated regions of tegumental cells are also known as cell bodies or tegumental cytons, where protein-synthesizing and sorting machinery (including endoplasmic reticulum and Golgi apparatus), is situated below the musculature and has connections to the syncytial surface part via cytoplasmic connections (Additional file 2: Figure S1a). Synthesized proteins, RNAs, and other cargos are transported to the cell surface by microtubule-lined cytoplasmic channels through cytoplasmic connections. The most common destination for mRNAs within the cell is in the immediate proximity of the site of translation, i.e. close to the endoplasmic reticulum. Therefore, mRNA would be expected in the tegumental cytons but not in the surface layer [62]. Nevertheless, mRNAs of SmPOP, SmTsp-2, and SmSP4 were localized not only in tegumental cytons, but a strong signal was also detected in tegumental tubercles (Figs. 3 and 5). These results suggest that a not fully understood transport mechanism exists for moving mRNA molecules from cytons to the schistosome surface. This cargo is usually transported through the tegument via extracellular vesicles sent to the outer environment. Recently, SmSP2, SmTsp-2, and Sm29 were identified in extracellular vesicles released from *Schistosoma* [63], which coincides with our localization in the tegumental tubercles.

The localization of mRNAs within eukaryotic cells is enormously diverse [64, 65]. The endoplasmic reticulum and Golgi apparatus provide well-established membrane-sorting machinery to shuttle mRNAs to distant regions within the cell [64]. These mRNAs are usually stored in granules and are ready for immediate or emergency use in a wide range of processes [64, 66]. They can (i) be reactivated, translated into proteins, and used in various rapid emergency processes including stress responses, metabolic reprogramming, repair of stress-induced damage, and adaptation to changed conditions; (ii) play a role in the repression of other mRNA species; or (iii) be directly sent for decay [67, 68]. We hypothesize that schistosomes may employ similar mechanisms, especially in such a dynamic structure as the tegument, which protects worms in the unfriendly blood environment of the host.

Antisense transcripts are frequently transcribed in eukaryotes and represent important regulators of gene expression; they control the state of chromatin or modulate the post-transcriptional fate of mRNAs [69, 70]. We identified antisense transcripts of SmPOP, SmSP5, and SmCB1 only, and they were localized exclusively in the oviduct (Additional file 3: Figures S2 and S5). As known from previous research, these antisense transcripts most likely do not encode proteins but may control gene expression [69].

## Conclusions

We validated and optimized a FISH method [23] for the detection of RNA transcripts in adult *S. mansoni* tissues. We documented the efficacy of the method by precise localization of the transcripts of selected proteins (SmCB1, SmPOP, SmTsp-2, and Sm29), whose distribution in schistosome adults was previously determined by other methods. In addition, we provided new insights into the localization of transcripts of these genes. Compared to RNAseq, FISH, due to its high sensitivity, is able to detect mRNA with low expression potential. The FISH methodology was then successfully applied to localize transcripts encoding serine proteases SmSP1 to SmSP5. Transcripts were found in *S. mansoni* females and males in various organs (parenchyma, tegument, reproductive organs) but with distinct patterns. Furthermore, we detected transcripts in previously unknown locations such as the syncytial part of the tegument or in tegumental tubercles. Based on the evidence of different transcript locations and our previous research [21, 22], we hypothesize that SmSPs may play various physiological roles in host–parasite interaction, including regulation of the host vascular system, repair of stress-induced damage, and/or adaptation to changed conditions in the external environment. However, elucidation of the precise function of individual SmSPs is a matter of future studies.

## Supplementary Information

**Additional file 1: Table S1.** Primers used to generate DIG-labelled RNA probes.

**Additional file 2: Figure S1.** Schematic representation of the adult *S. mansoni* surface and detailed micrograph of SmTsp-2 localization in the tegument and parenchyma of *S. mansoni* adult males.

**Additional file 3: Figure S2.** Localization of antisense mRNA of SmCB1, SmPOP, SmTsp-2 and Sm29 in adult *S. mansoni* females using FISH. **Figure S3.** Localization of antisense mRNA of SmCB1, SmPOP, SmTsp-2 and Sm29 in adult *S. mansoni* males using FISH. **Figure S4.** FISH with the probe for bacterial (*neo*) gene and a negative control (with no probe) in *S. mansoni* males and females. **Figure S5.** Localization of antisense mRNA of SmSP1 to SmSP5 in adult *S. mansoni* females using FISH. **Figure S6.** Localization of antisense mRNA of SmSP1 to SmSP5 in adult *S. mansoni* males using FISH.

## Data Availability

All data generated or analyzed during this study are included in this published article and its supplementary information files.

## Abbreviations

DIG: Digoxigenin
FISH: Fluorescence in situ hybridization
HRP: Horseradish peroxidase
PBS: Phosphate-buffered saline
SSC: Standard saline citrate
SmSP1-5: *S. mansoni* serine protease 1–5
SmCB1: *S. mansoni* cathepsin B1
Sm29: Membrane-bound glycoprotein found on *S. mansoni* tegument, GenBank accession number AAC98911.1
SmPOP: *S. mansoni* prolyl oligopeptidase
SmTsp-2: *S. mansoni* tetraspanin 2
