# Supplementary material for: Spatial expression pattern of serine proteases in the blood fluke Schistosoma mansoni determined by fluorescence RNA in situ hybridization
Source: Parasit Vectors. 2021 May 22;14:274. doi: 10.1186/s13071-021-04773-8 (PMC8140508; doi:10.1186/s13071-021-04773-8)
Supplement: Supplementary file 1 — Additional file 1: Table S1. Primers used to generate DIG-labelled RNA probes. [file 13071_2021_4773_MOESM1_ESM.pdf]

Table S1 Primers used to generate DIG-labeled RNA probes.

Name	GenBank ID	Probe length [nt]	Forward primer (5´→ 3´)	Reverse primer (5´→ 3´)
**SmSP1**	KF535923	685	AAAAGGTGCTTGGGCTTTTA	GCCCCATGATACAATTCCAG
**SmSP2**	KF510120	1503	CACCATGATCTTAATCAATACATACTT AATCTACTTTATATGTTTT	AAATATTTTTGTTGCTAATTGTTTTGATA TCCAA
**SmSP3**	KF510121	623	ATGGCCATGGATGGTATCAG	CAGGCACAGCACATGATTTC
**SmSP4**	KF510122	930	CCCTGAACATTGTGGTCGTA	TAACCTGCTTTACCGCATCC
**SmSP5**	KF939306	551	CTGGACATTGTGCATGTGGT	CCAACCATTTCCAGCTATTGA
**SmCB1**	AJ506157	872	AAGCAACCGTTTCCACTCAC	TAATTCGACCGGCTGTTACC
**SmPOP**	KF956809	911	CCGGATCAACCTACTTGGAA	GCTTGGAAATCATCGAAGGA
**SmTsp-2**	AF521091	713	CTCTTCGTTGTGGGTATAAG	CATGTTCGTCATTACGGTAC
**Sm29**	AAC98911	752	TTTCTCGGAATTG	TCCATACACATTCACATA
