# Supplementary material for: Spatial expression pattern of serine proteases in the blood fluke Schistosoma mansoni determined by fluorescence RNA in situ hybridization
Source: Parasit Vectors. 2021 May 22;14:274. doi: 10.1186/s13071-021-04773-8 (PMC8140508; doi:10.1186/s13071-021-04773-8)
Supplement: Supplementary file 2 — Additional file 2: Figure S1. Schematic representation of the adult S. mansoni surface and detailed micrograph of SmTsp-2 localization in the tegument and parenchyma of S. mansoni adult males. [file 13071_2021_4773_MOESM2_ESM.pdf]

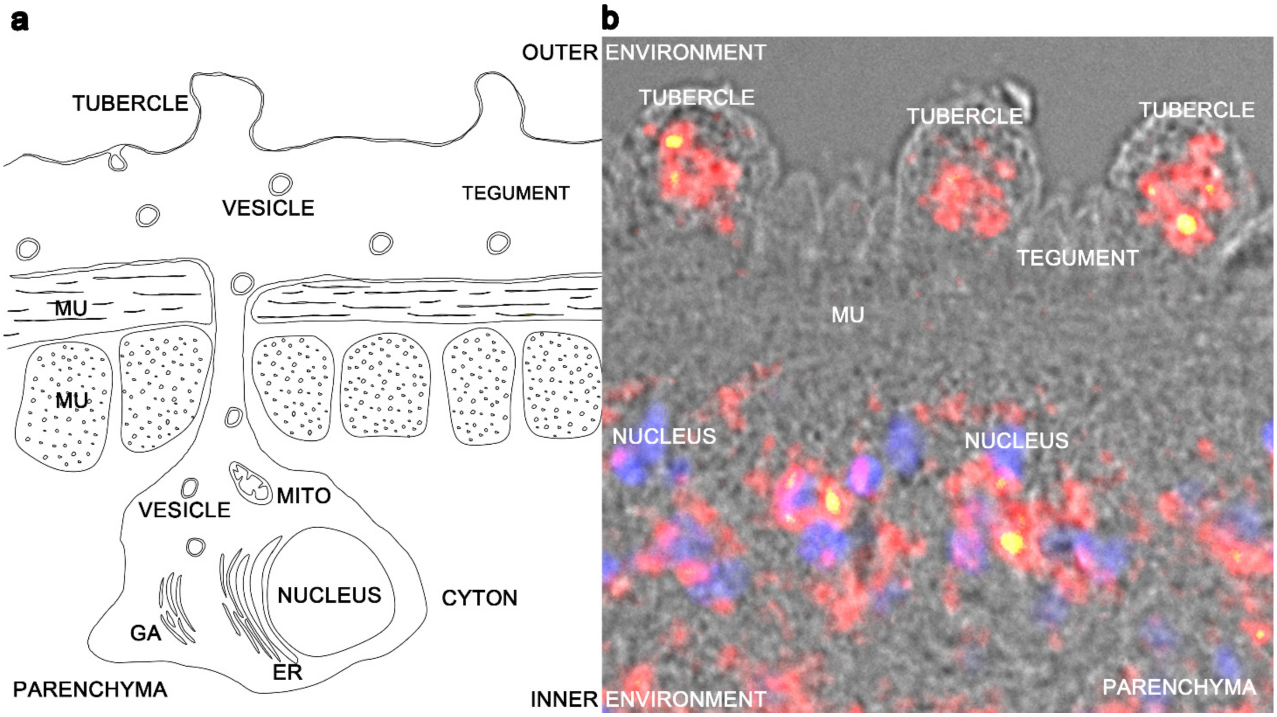

Figure S1 Schematic representation of the adult *S. mansoni* surface and detailed micrograph of SmTsp-2 localization in tegument and parenchyma of *S. mansoni* adult male. **a** Diagrammatic representation of the adult schistosome tegument and surrounding tissues. Replication, transcription and translation apparatus are present exclusively in tegument cell-body (called cyton) located beneath the muscle layers. Cargo like proteins or RNAs are transported to through cytoplasmatic connection to the tegumental surface in membranous vesicles. **b** The tissue section of adult *S. mansoni* male was probe with DIG-labelled RNA probes designed to detect mRNA of SmTsp-2. Hybridized transcripts were visualized tyramide amplification assay (red). DAPI was used to label cell nuclei (blue). Image show merged fluorescent channels and differential interference contrast. ER – endoplasmatic reticulum, GA – Golgi apparatus, MITO – mitochondria, MU – muscle.
