# Supplementary material for: Spatial expression pattern of serine proteases in the blood fluke Schistosoma mansoni determined by fluorescence RNA in situ hybridization
Source: Parasit Vectors. 2021 May 22;14:274. doi: 10.1186/s13071-021-04773-8 (PMC8140508; doi:10.1186/s13071-021-04773-8)
Supplement: Supplementary file 3 — Additional file 3: Figure S2. Localization of antisense mRNA of SmCB1, SmPOP, SmTsp-2 and Sm29 in adult S. mansoni females using FISH. Figure S3. Localization of antisense mRNA of SmCB1, SmPOP, SmTsp-2 and Sm29 in adult S. mansoni males using FISH. Figure S4. FISH with the probe for bacterial (neo) gene and a negative control (with no probe) in S. mansoni males and females. Figure S5. Localization of antisense mRNA of SmSP1 to SmSP5 in adult S. mansoni females using FISH. Figure S6. Localization of antisense mRNA of SmSP1 to SmSP5 in adult S. mansoni males using FISH. [file 13071_2021_4773_MOESM3_ESM.pdf]

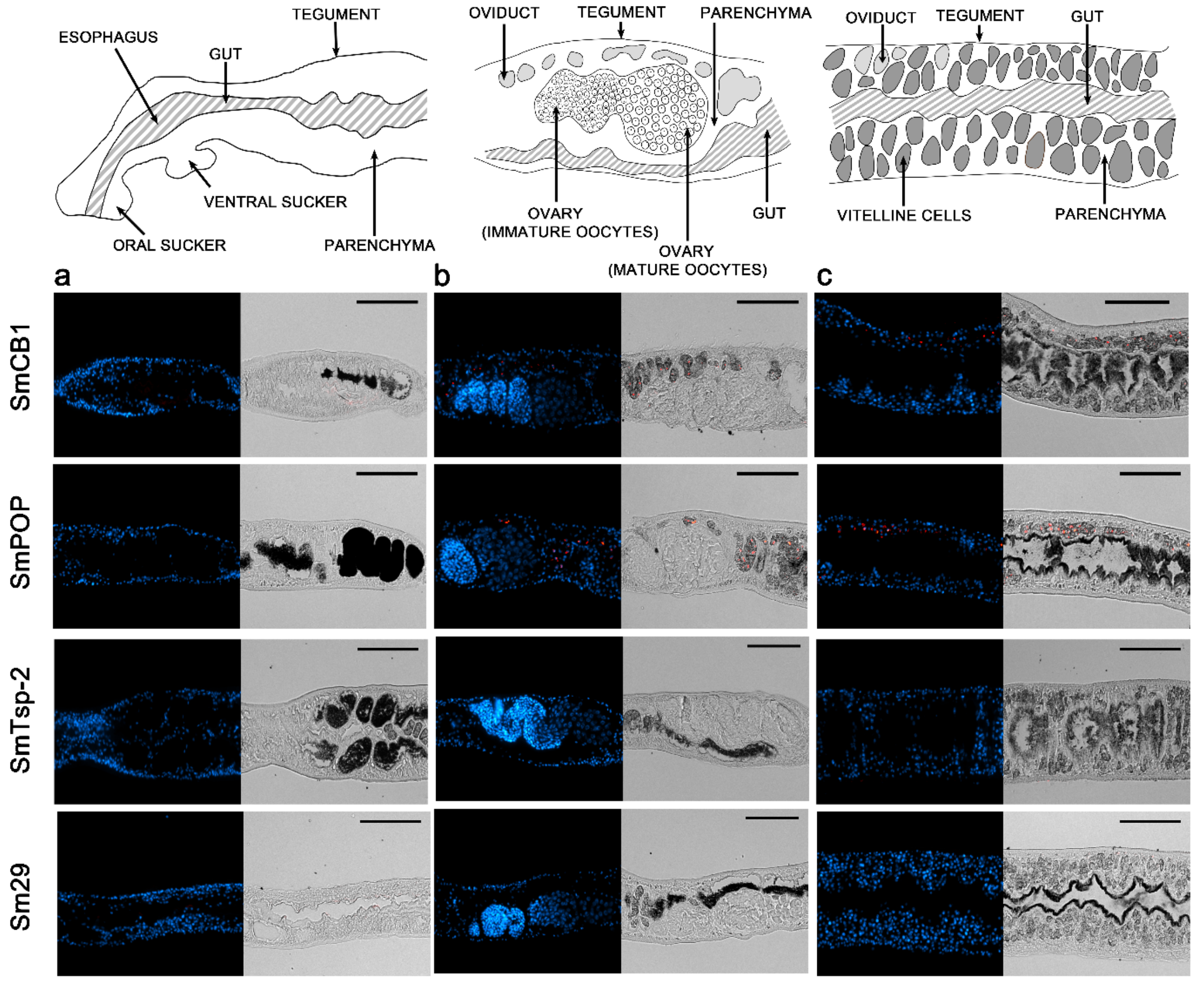

Figure S2 Localization of anti-sense mRNA of SmCB1, SmPOP, SmTsp-2 and Sm29 in adult *S. mansoni* females using FISH. Semi-thin (6 µm) sections of adult *S. mansoni* female worms were probed with DIG-labelled RNA probes designed to detect SmCB1, SmPOP, SmTsp-2 or Sm29 anti-sense mRNAs. Probes hybridized with transcripts were visualized by tyramide amplification assay (red). Adult females were monitored in three parts: **a** an anterior part, **b** oviduct, mature and immature ovaries, and **c** vitellaria, gut, and oviduct (if present on the section). DAPI was used to label nuclear DNA (blue). The left columns show merged fluorescent channels; in the right columns, the fluorescent red signal is merged with differential interference contrast. Only the anti-sense transcripts of SmCB1 and SmPOP (red) are present in oviduct. The scale bars represent 100 µm.

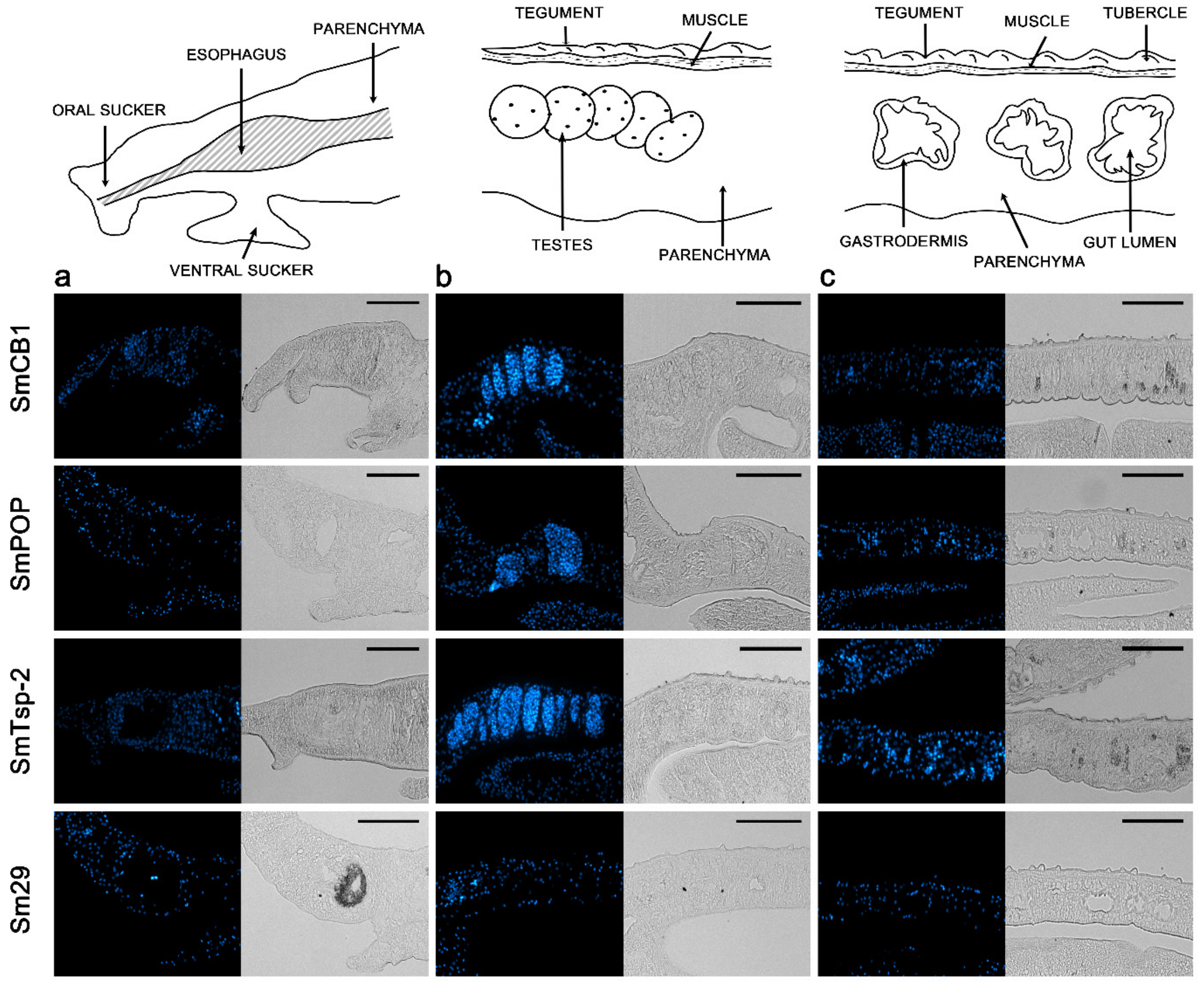

Figure S3 Localization of anti-sense mRNA of SmCB1, SmPOP, SmTsp-2 and Sm29 in adult *S. mansoni* males using FISH. Semi-thin (6 μm) sections of adult *S. mansoni* male worms were probed with DIG-labelled RNA probes designed to detect SmCB1, SmPOP, SmTsp-2 or Sm29 anti-sense mRNAs. Probes hybridized with transcripts were visualized by tyramide amplification assay (red). Adult males were monitored in three parts: **a** a head part, **b** testes, and **c** a posterior part with the focus on parenchyma, tegument and gut. DAPI was used to label nuclear DNA (blue). The left columns show merged fluorescent channels; in the right columns, the fluorescent red signal is merged with differential interference contrast. No anti-sense transcripts of studied genes were observed. The scale bars represent 100 μm.

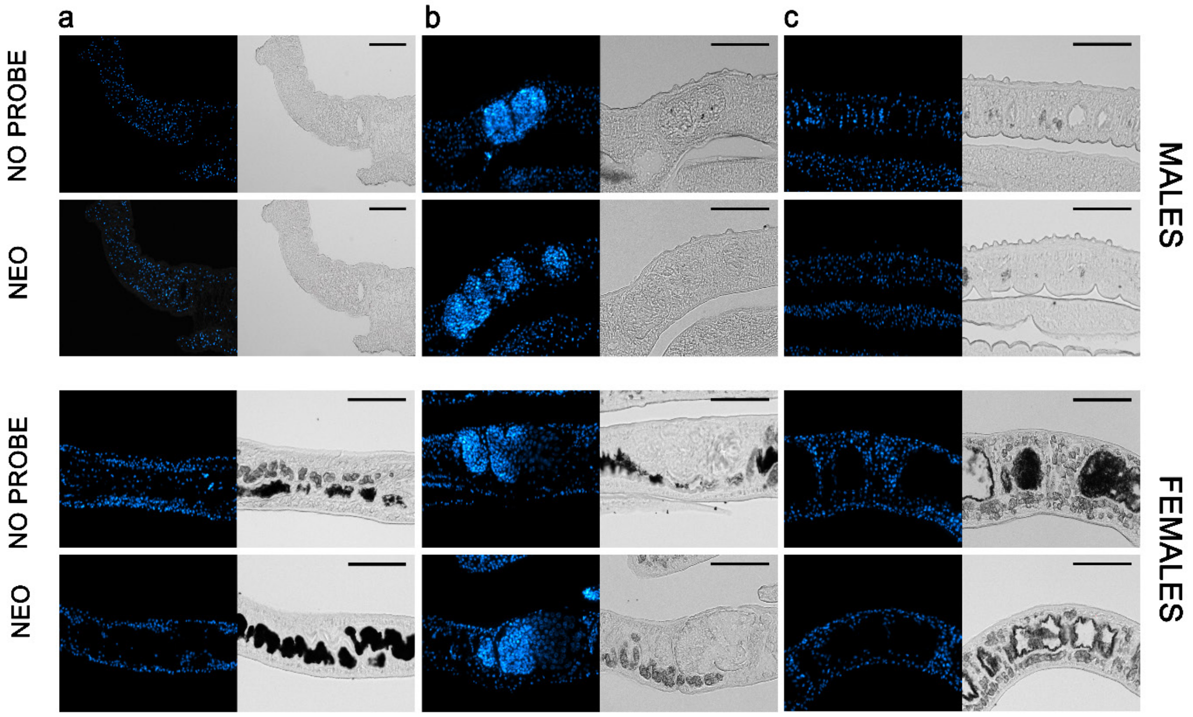

Figure S4 FISH with the probe for bacterial (neo) gene and a negative control (with no probe) in *S. mansoni* males and females. Semi-thin (6 µm) sections of adult male and female worms were probed using DIG-labelled RNA probe designed to detect bacterial neomycin gene (NEO) or with hybridization buffer instead of the probe (NO PROBE). Probes hybridized with transcripts were visualized by tyramide amplification assay (red). Adult worms were monitored in three parts: **a** an anterior part of the female or head of the male, **b** oviduct, mature and immature oocytes of females or testes of males, **c** vitellaria and gut of female or parenchyma, tegument and the gut of the male. DAPI was used to label nuclear DNA (blue). The left columns show merged fluorescent channels; in the right columns, the fluorescent red signal is merged with differential interference contrast. No fluorescent red signal was observed during both control experiments. The scale bars represent 100 µm.

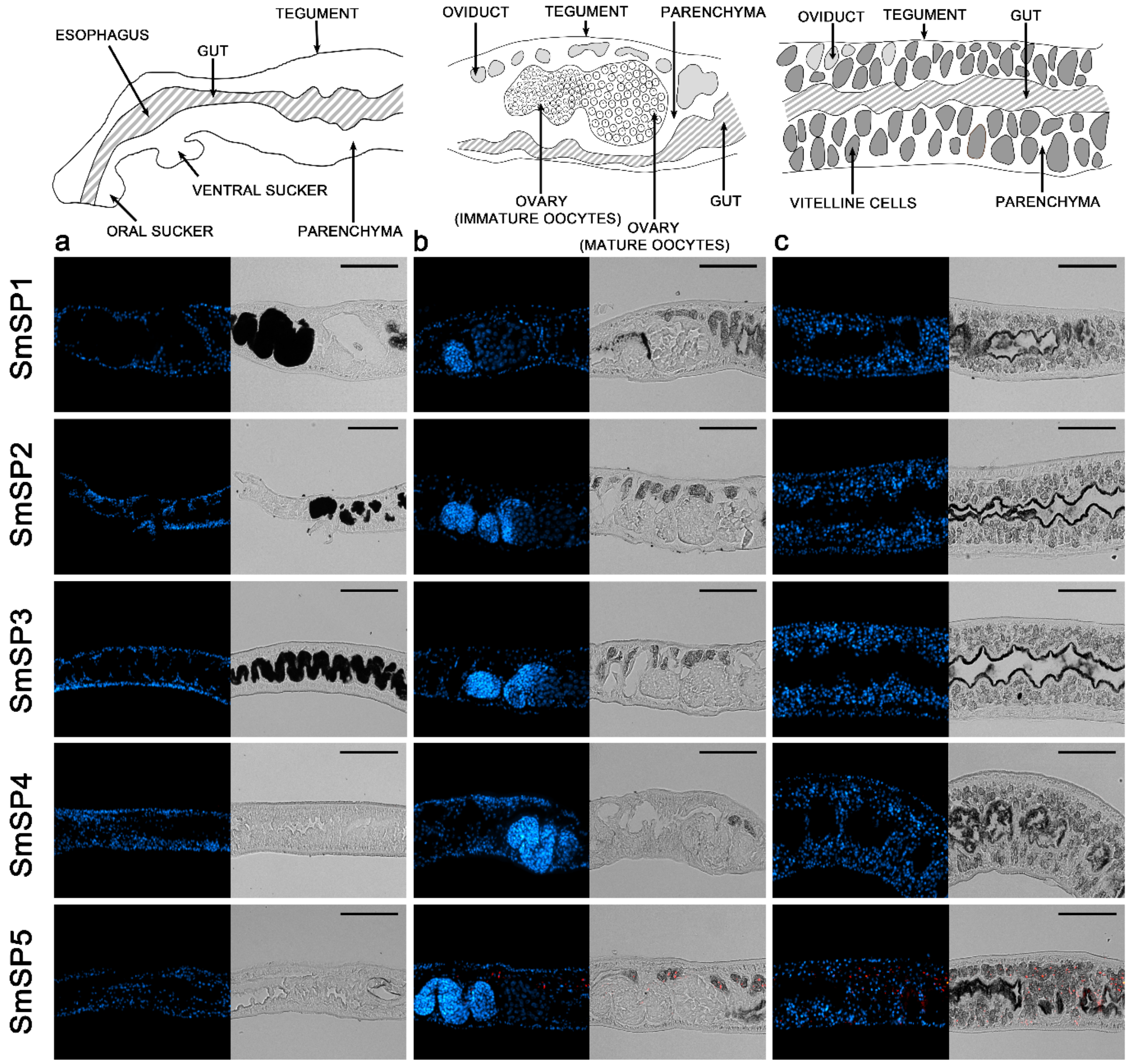

Figure S5 Localization of anti-sense mRNA of SmSP1 to SmSP5 in adult *S. mansoni* females using FISH. Semi-thin (6 µm) sections of adult *S. mansoni* female worms were probed with DIG-labelled RNA probes designed to detect mRNAs of SmSP1 to SmSP5. Probes hybridized with transcripts were visualized by tyramide amplification assay (red). Adult females were monitored in three parts: **a** an anterior part, **b** oviduct, mature and immature oocytes, and **c** vitellaria, gut and oviduct (if present on the section). DAPI was used to label nuclear DNA (blue). The left columns show merged fluorescent channels; in the right columns, the fluorescent red signal is merged with differential interference contrast. The only identified anti-sense transcripts (red) were for the SmSP5 sense probe in oviduct. The scale bars represent 100 µm.

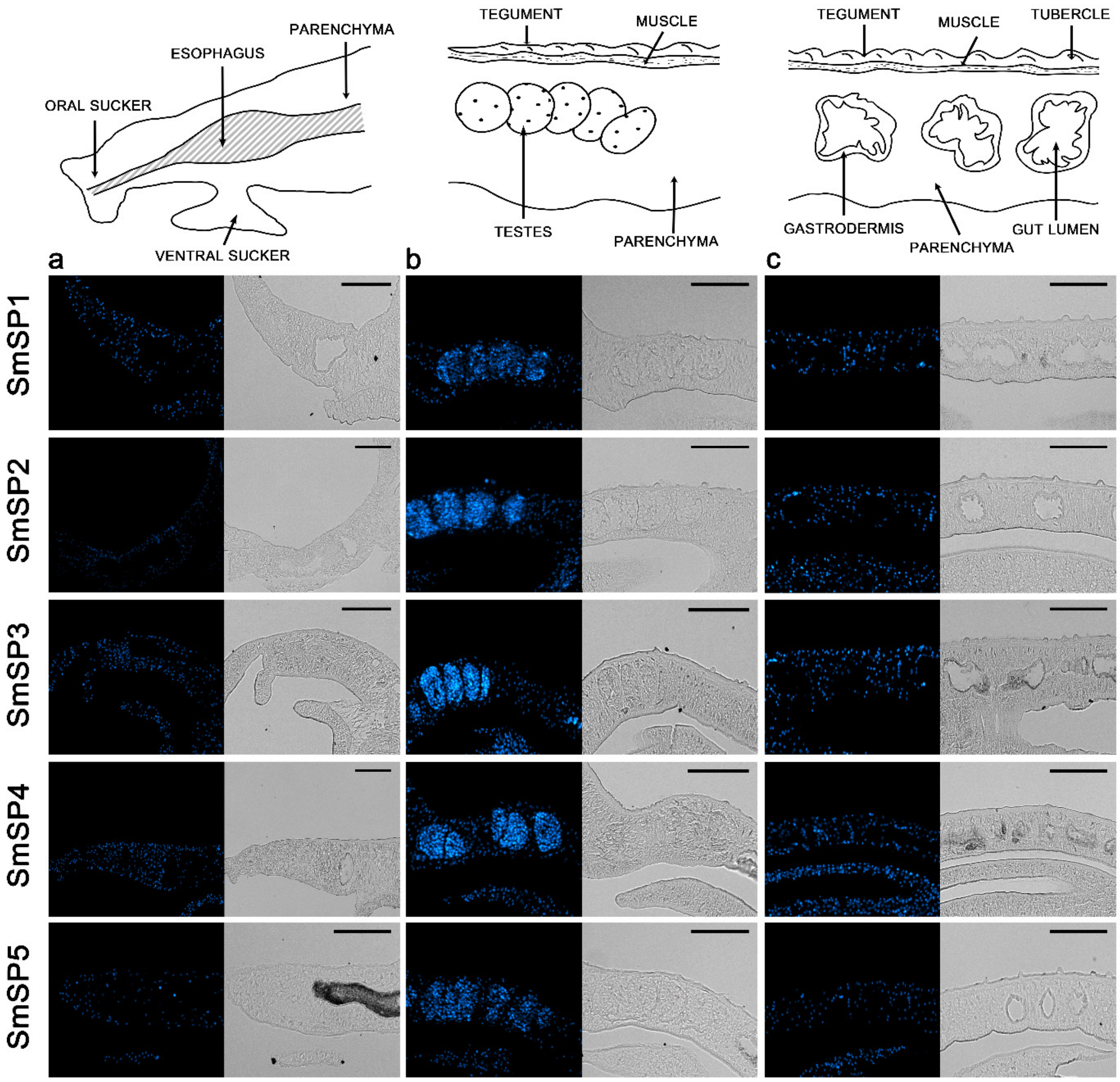

Figure S6 Localization of anti-sense mRNA of SmSP1 to SmSP5 in adult *S. mansoni* males using FISH. Semi-thin (6 µm) sections of adult *S. mansoni* male worms were probed with DIG-labelled RNA probes designed to detect mRNAs of SmSP1 to SmSP5. Probes hybridized with transcripts were visualized by tyramide amplification assay (red). Adult males were monitored in three parts: **a** a head part, **b** testes, and **c** a posterior part with the focus on parenchyma, tegument and gut. DAPI was used to label nuclear DNA (blue). The left columns show merged fluorescent channels; in the right columns, the fluorescent red signal is merged with differential interference contrast. No anti-sense transcripts of studied genes were observed. The scale bars represent 100 µm.
